# Soy product intake and risk of incident disabling dementia: the JPHC Disabling Dementia Study

**DOI:** 10.1007/s00394-022-02937-5

**Published:** 2022-07-05

**Authors:** Utako Murai, Norie Sawada, Hadrien Charvat, Manami Inoue, Nobufumi Yasuda, Kazumasa Yamagishi, Shoichiro Tsugane, For the JPHC Study Group

**Affiliations:** 1 Division of Cohort Research, National Cancer Center Institute for Cancer Control, 5-1-1 Tsukiji, Chuo-ku, Tokyo, 104-0045 Japan; 2 Division of International Health Policy Research, National Cancer Center Institute for Cancer Control, Tokyo, Japan; 3 Faculty of International Liberal Arts, Juntendo University, Tokyo, Japan; 4 Division of Prevention, National Cancer Center Institute for Cancer Control, Tokyo, Japan; 5 Department of Public Health, Kochi University Medical School, Kochi, Japan; 6 Department of Public Health Medicine, Faculty of Medicine, and Health Services Research and Development Center, University of Tsukuba, Tsukuba, Japan; 7 National Institute of Health and Nutrition, National Institutes of Biomedical Innovation, Health and Nutrition, Tokyo, Japan

**Keywords:** Soy products, Fermented soy, Disabling dementia, Cohort study, Japan

## Abstract

**Purpose:**

We evaluated the association between total soy, soy product (natto, miso and tofu) and isoflavone intake and incident disabling dementia in a Japanese population.

**Methods:**

We conducted a population-based prospective study in 18,991 men and 22,456 women. Intake of soy products and isoflavone was calculated using a validated food frequency questionnaire when participants were 45–74 years old (1995 and 1998). Incident disabling dementia was defined by the daily living disability status related to dementia in the long-term care insurance program of Japan from 2006 to 2016. Multivariate hazard ratios (HRs) and 95% confidence intervals (CIs) of disabling dementia were calculated by quintiles of total soy, individual soy product and isoflavone intake, using Cox proportional hazard regression models.

**Results:**

Total soy product intake was not associated with disabling dementia risk in both men and women. By individual soy products, natto intake was marginally inversely associated with disabling dementia in women (trend *P* = 0.050). When we stratified by age, this inverse association was clearer in women aged under 60 years (multivariate HR for the highest versus lowest quintile was 0.78, 95% CI 0.59–1.04, trend *P* = 0.020 for those aged under 60 years and 0.90, 95% CI 0.77–1.05, trend *P* = 0.23 for those aged 60 years and older, respectively). Any soy product or isoflavone intake was not associated with disabling dementia risk in men.

**Conclusions:**

Although total soy product intake was not associated with disabling dementia risk, natto intake may contribute to reducing the risk of disabling dementia in women, especially in those aged under 60 years.

**Supplementary Information:**

The online version contains supplementary material available at 10.1007/s00394-022-02937-5.

## Introduction

With the worldwide aging of the population, dementia is a growing global problem. 55 million people currently have dementia, and around 10 million new cases are diagnosed every year [1]. Dementia is related to lifestyle, including eating habits [2], and therefore subject to prevention.

Soy products are widely consumed in Asia [3]. They are rich in isoflavones, which may protect cognitive function [4] or Alzheimer's disease (AD) [5]. A previous meta-analysis study reported that isoflavone intake improved cognitive function and memory in randomized controlled trials [6]. Therefore, we hypothesized that soy product intake may decrease the risk of disabling dementia. So far, several studies have examined the association between soy product intake and risk of cognitive function. High soy product intake was associated with decreased risk of cognitive impairment in prospective cohort studies [7, 8], while another study found no association in cross-sectional study [9]. Regarding dietary patterns, a healthy dietary pattern which included soy products was associated with decreased dementia risk in elderly Japanese [10]. Among individual soy products, high tofu intake increased [11, 12] or decreased [13] the risk of cognitive impairment, whereas fermented soy product intake decreased [13, 14] or was not associated [8] with risk. Regarding fermented soy products eaten in Japan, natto contains bioactive components, such as polyamine [15] and nattokinase [16]: polyamine (spermidine) has been shown to protect against neurodegeneration and cognitive decline [17], and nattokinase protects the hippocampus against β-amyloid-induced neuronal damage [18, 19] in animal experiments. In addition, the Japan Public Health Center-Based Prospective Study (JPHC Study) showed that natto intake was inversely associated with risk of hypertension or stroke [20, 21], which are considered risk factors of dementia. Consumption of soy products, particularly natto, may therefore have protective effects against dementia. The only prospective study from Japan reported that natto intake was not associated with cognitive impairment [8]. However, the investigation area in that study was limited to a single prefecture and the participants were a relatively small number of elderly people.

Accordingly, the association between soy product intake and cognitive function remains inconsistent. In addition, to our knowledge, no study has investigated the association between soy product intake and risk of incident dementia.

We collected a large number of disabling dementia cases from the certification of Japan’s national long-term care insurance (LTCI) system. We defined the outcome of this study as dementia that required care (disabling dementia) under the LTCI system, which provides national compulsory insurance for all individuals aged 40 years or older in Japan. Disabling dementia was assessed not only from cognitive impairment but also by a comprehensive review of health condition, including evaluation by a primary care physician.

Here, we used this information to evaluate the association of total soy and individual soy product intake and disabling dementia in a middle-aged Japanese population, in whom consumption of soy products is high compared to Western countries.

## Materials and methods

### Study population

The JPHC Study consists of two subcohorts: cohort I [started in 1990, five public health centers (PHCs) of Iwate, Akita, Nagano, Okinawa and Tokyo prefectures, and participants aged 40–59 years], and cohort II (started in 1993, six PHCs of Ibaraki, Niigata, Kochi, Nagasaki, Okinawa and Osaka prefectures, and participants aged 40–69 years). Details of the study design have been described elsewhere [22, 23]. The JPHC study is consisted of 140,420 Japanese participants (68,722 men and 71,698 women). We conducted surveys at baseline, 5- and 10-year follow-up, in which participants were asked to complete self-administered questionnaires about their lifestyle and medical history. Because the 5-year follow-up survey included a more detailed food frequency questionnaire (FFQ) than the baseline survey, we included participants who responded to the 5-year follow-up survey in this analysis. Participants were informed of the objectives of the study, and that completion of the survey questionnaire was regarded as providing consent to participate. The study was approved by the National Cancer Center Japan institutional review board (Number: 2001-021, 2015-085, 2015-312).

This study included 62,401 participants from eight areas in which follow-up data on disabling dementia were available, namely Omonogawa and Yokote districts in Yokote city in Akita prefecture; Tomobe district in Kasama city and Iwase district in Sakuragawa city in Ibaraki prefecture; Usuda district in Saku city in Nagano prefecture; Kagami and Noichi districts in Konan city in Kochi prefecture; and Gushikawa district in Uruma city in Okinawa prefecture.

### Disabling dementia cases and follow-up

We defined the outcome as dementia that required care (disabling dementia) under the LTCI system, which is administered by the Ministry of Health, Labor and Welfare Bureau of Japan. The LTCI system was initiated in 2000 for residents aged 65 years and older and for those with disabilities aged 40–64 years, and is administered by municipalities. The municipality assesses applications using two documents, namely a comprehensive assessment of the applicant’s functional health condition, and a written opinion about the applicant’s disability status by a primary care physician. We defined disabling dementia as certification of any level of need for long-term care, and a written opinion of the grade of cognitive disability (grade IIa, IIb, IIIa, IIIb, IV or M). Grades of cognitive disability were no dementia; some dementia but almost independent in daily life (level I); dementia with some difficulty in communicating but with independence in daily living with minimal observation (level IIa and IIb); dementia with some difficulty in communicating and a need for partial care (level IIIa and IIIb); severe dementia with difficulty in communicating and a need for complete care (level IV); and significant psychiatric symptoms, behavioral problems or serious physical illness and need for specialized medical care (M) [24]. These criteria of disabling dementia were validated against dementia diagnoses by neuropsychiatrists: the sensitivity was moderate (73%), and the specificity was high (96%) [25]. Additionally, grades of cognitive disability have been well-correlated with the Mini-Mental State Exam (MMSE) score (Spearman rank correlation coefficient − 0.736) [26].

In this study, certification records in the LTCI system were available from 1 January 2006 to 31 December 2016. Therefore, the follow-up start point of this study was defined as January 1, 2006. We followed eligible participants until 31 December 2016.

### Soy product intake

We used the JPHC Study food frequency questionnaire (FFQ) data from the 5-year follow-up survey conducted in 1995–1998. The survey included questions about the frequency and portion size of about 138 food and beverage items [27, 28]. Standard portion sizes for each food item were small (50% less than the standard serving size), medium (proportional to standard serving size), and large (50% larger than the standard serving size). The nine frequency categories for each food item were never, 1–3 times/month, 1–2 times/week, 3–4 times/week, 5–6 times/week, 1 time/day, 2–3 times/day, 4–6 times/day, and ≥ 7 times/day; and for drink items were never, 1–2 times/week, 3–4 times/week, 5–6 times/week, 1 time/day, 2–3 times/day, 4–6 times/day, 7–9 times/day, and ≥ 10 times/day. The dietary intake of each food item was calculated by multiplying frequency by the standard or relative portion sizes for each food item, excluding soy milk, green tea and miso: intake of soymilk and green tea was calculated by multiplying frequency by the standard portion, and because miso is a seasoning used in miso soup and accounts for 8% of the amount of miso soup, miso intake was calculated by multiplying the amount of miso soup by 0.08. The Spearman’s correlation coefficients between the energy-adjusted intake of total soy products from the FFQ and those from dietary records were 0.53 for men and 0.49 for women in cohort I [29], and 0.52 and 0.54 in cohort II [30]. The amount of total soy products was the sum of tofu, yushidofu, koya-dofu, abura-age, natto, miso, and soymilk items from the FFQ. Tofu is the sum of tofu, yushidofu and koya-dofu items from the FFQ. The amount of total, individual soy products and isoflavones was adjusted for total energy intake using the residual method [31]. Because soy products are isoflavone-rich, we also calculated total isoflavone intake, including daidzein and genistein. Daidzein and genistein intakes were estimated using the values from each soy product in a specially developed food composition table for isoflavones in Japanese foods [32, 33]. The Spearman’s correlation coefficients between the energy-adjusted intake of isoflavone from the FFQ and those from dietary records were 0.65 for men and 0.56 for women in cohort I, and 0.53 and 0.45 in cohort II, respectively (unpublished data).

### Statistical analysis

We included 62,401 participants in this study. We excluded non-Japanese participants (*n* = 51), as well as those with an incorrect birth date (*n* = 3), duplicate registration (*n* = 4), refusal to follow-up (*n* = 11), and those who moved out or died before the start of follow-up (*n *= 11,656), leaving 50,676 participants as eligible for analysis. We further excluded those who did not answer the 5-year follow-up questionnaire survey (*n* = 6637), those who left the frequency of soy products intake unanswered (*n *= 414), and those who reported total energy intake at the upper or lower 2.5% ends of the range (1030 and 4169 kcal for men and 882 and 3640 kcal for women, respectively). Finally, 41,447 participants (18,991 men and 22,456 women) were included in the analysis (Supplemental Fig. 1).

We divided participants into quintiles of intake for total soy and individual soy products by sex, except for natto only in men, and used the lowest quintile group as reference. Regarding the 4,208 men who answered that they did not eat natto, we categorized these into the first fifth, and divided the remaining men into quartiles. Sex-specific, mean values and prevalence of selected factors were calculated and compared by total soy product intake using linear or logistic regression analyses. Dietary intake was adjusted for energy intake using the residual method except for total energy and green tea, and P values were calculated from the median value of each food by total soy product intake using the Kruskal–Wallis test.

Models were adjusted for confounding factors. Model 1 was adjusted for age (continuous) and stratified by 8 areas. Model 2 was additionally adjusted for body mass index (< 18.5, 18.5–24.9, 25.0–29.9, or ≥ 30 kg/m^2^, missing), smoking status (never, former, < 20 cigarettes/day, and ≥ 20 cigarettes/day, missing), alcohol intake (0, 1–149, 150–299, or ≥ 300 ethanol g/week, missing), total metabolic equivalents (hours per day, quartiles, missing), history of cancer (yes or no), history or medication for diabetes (yes or no), medication for hypertension or hypercholesterolemia (yes or no), menopausal status (yes, no, missing; women only), health checkup in the past year (yes or no), job status (employed, no, missing), living status (living with others, alone, missing) and dietary intakes of total energy (quintiles), vegetables (quintiles), fruits (quintiles), fish (quintiles), green tea (quintiles) and sodium (quintiles). Missing values for dietary intake excluding total energy were given a value of 0. We created dummy variables for covariates and missing data. Trends were calculated by assigning median value to categories of each soy product intake. Additionally, as participants in the previous studies from Japan [8, 10] which reported associations between soy products and cognitive function or dementia were aged 60 years and older, we stratified participants by age at the time of the 5-year questionnaire follow-up survey. As a supplementary analysis, to consider types of dementia, disabling dementia cases were classified into two categories: with or without a history of stroke, following systematic stroke registration confirmed according to the criteria of the National Survey of Stroke [34] as well as self-reports at the baseline, 5- and 10-year follow-up questionnaires. The end of the follow-up period was set for the end of 2009 for Yokote, Saku and Uruma areas, and for the end of 2012 for Kasama, Sakuragawa and Konan areas, with consideration to the availability of stroke registry data. In addition, using the dataset of a supplementary analysis, we examined the mediating role of stroke incidence between soy product intake and disabling dementia risk the follow-up period (2009 or 2012). These analyses were carried out using multistate models, an extension of competing risk survival analysis, which allows the analysis of various transition rates from an initial state to intermediate events and subsequently to final states in the same framework [35]. In particular, these models were used to simultaneously estimate the risk associated with soy product intake in three transitions: (1) from healthy state to incident dementia in those free of incident stroke; (2) from healthy state to incident stroke; and (3) from incident stroke to incident dementia. Age was used as the follow-up starting point (2006 or 2009), and the analyses were adjusted for covariates in multivariate-adjusted models.

Person years of follow-up were calculated for each participant from the follow-up start point until the date of diagnosis of disabling dementia, migration from a study area to a non-study area, date of death, or the end of follow-up, whichever occurred first. Cox proportional hazards models were used to estimate hazard ratios (HRs) and 95% confidence intervals (CIs).

We confirmed the proportional hazards assumption according to total soy product intake × time interactions and found that the assumption was not violated. All probability values for the statistical tests were 2-tailed, and probability values below 0.05 were considered significant. For each set of analyses shown in the tables of results, multiple comparison was accounted for by Bonferroni correction. All statistical analyses were performed using the SAS software, version 9.4. (SAS Institute, Cary, NC, USA) and R software, version × 64 4.1.2; in particular, the multistate model analyses were performed using the “mstate” package.

## Results

During a mean follow-up of 9.4 years, 4911 cases of disabling dementia (1996 men and 2915 women, respectively) were observed. The baseline characteristics of participants according to total soy product intake are shown in Table 1. Participants who consumed soy products tended to be older, have a higher proportion of treatment for hypertension or hypercholesterolemia and consume more vegetables in both men and women.

**Table 1:** Basic characteristics of participants among 18,991 men and 22,456 women according to quintile of total soy product intake

Total soy product intake	Men					*P* for difference	Women					*P* for difference
	Q1	Q2	Q3	Q4	Q5		Q1	Q2	Q3	Q4	Q5	
Number at risk	3,798	3,798	3,799	3,798	3,798		4,491	4,491	4,492	4,491	4,491	
Age at 5-year follow-up survey, mean	54.5	55.3	55.8	56.3	57.0	< 0.001	55.4	56.2	56.5	56.7	57.4	< 0.001
Age at baseline, mean	63.9	64.9	65.5	66.0	66.7	< 0.001	64.8	65.7	66.2	66.4	67.3	< 0.001
Body mass index, kg/m^2^	23.5	23.4	23.5	23.6	23.7	< 0.001	23.3	23.3	23.4	23.5	23.7	< 0.001
Current smokers, %	71.7	70.2	69.3	64.9	65.0	< 0.001	16.0	12.4	12.2	11.1	11.7	< 0.001
Ethanol intake ≥ 300 g/week, %	36.5	34.6	31.4	30.7	26.7	< 0.001	2.1	1.2	0.8	0.7	0.7	< 0.001
Physical activities, mean (SD), MET-h/day	33.5	33.6	33.5	33.6	33.5	0.93	32.5	32.5	32.6	32.6	32.5	0.63
Medication for hypertension, %	14.5	16.8	17.9	19.9	21.6	< 0.001	15.2	19.0	19.8	20.7	22.0	< 0.001
Medication for hypercholesterolemia, %	2.9	3.4	4.4	4.9	5.6	< 0.001	5.7	7.9	7.7	9.7	10.4	< 0.001
History of diabetes mellitus^a^, %	5.4	5.8	6.2	6.9	8.9	< 0.001	3.2	3.1	2.9	3.7	4.8	< 0.001
Health checkup in the past year, %	80.8	86.0	85.9	88.3	85.8	< 0.001	84.3	87.8	87.9	89.0	88.5	< 0.001
Employed, %	88.2	88.0	87.5	85.8	84.3	< 0.001	59.3	51.8	48.8	46.7	44.7	< 0.001
Living with others, %	96.3	97.8	98.1	98.1	97.2	< 0.001	93.5	95.4	96.1	97.2	95.8	< 0.001
Menopause, %	–	–	–	–	–	–	69.1	74.2	76.5	78.3	82.0	< 0.001
Dietary intake^b^, median												
Total energy, kcal/day	2079	2125	2133	2119	2018	< 0.001	1786	1807	1833	1811	1753	< 0.001
Vegetables, g/day	123.9	153.4	172.2	181.8	197.3	< 0.001	160.8	191.3	206.9	218.1	229.1	< 0.001
Fruits, g/day	113.0	139.7	153.2	163.2	162.6	< 0.001	194.7	219.9	219.8	228.4	219.5	< 0.001
Fish, g/day	70.9	80.0	83.3	88.0	83.3	< 0.001	74.5	81.2	84.0	85.5	80.7	< 0.001
Green tea, g/day	300	325	360	394	360	< 0.001	326	420	600	600	600	< 0.001
Sodium, g/day	9.1	11.0	12.0	12.6	13.2	< 0.001	9.4	10.8	11.7	12.2	12.9	< 0.001
Total soy products^c^, g/day	30.1	52.0	70.9	94.1	141.8	< 0.001	31.0	51.9	70.3	93.0	142.5	< 0.001
Natto, g/day	2.0	7.4	13.1	19.9	26.3	< 0.001	2.8	8.8	14.5	21.3	26.4	< 0.001
Miso^d^, g/day	7.8	16.7	21.3	22.9	23.6	< 0.001	6.9	12.6	17.3	19.8	21.2	< 0.001
Tofu^e^, g/day	13.8	23.2	30.3	43.1	74.2	< 0.001	14.8	25.3	32.6	45.2	76.6	< 0.001
Isoflavone^f^, mg/day	14.0	26.5	37.9	51.3	73.1	< 0.001	14.5	27.1	38.1	50.8	72.8	< 0.001

Sex-specific, age-adjusted mean values and prevalence of variables were calculated and compared by total soy product intake using linear or logistic regression analyses

^a^History of diabetes mellitus included participants who reported having diabetes and/or those who answered that they used diabetes medication

^b^Food intakes were adjusted for energy intake using the residual method except for total energy and green tea. *P* values were calculated with the Kruskal–Wallis test

^c^Sum of the amount of tofu, yushidofu, koya-dofu, deep-fried tofu (abura-age), natto, miso and soy milk

^d^Amount of miso was calculated from the amount of miso soup

^e^Sum of the amount of tofu, yushidofu and koya-dofu

^f^Sum of the amount of genistein and daidzein

We observed no association between total soy product intake and disabling dementia in both men and women: the multivariate HRs (95% CI) for the highest versus lowest quintile were 1.07 (95% CI 0.92–1.24, *P* for trend = 0.25) for men and 1.04 (95% CI 0.92–1.18, *P* for trend = 0.44) for women (Table 2). Among individual soy products, natto intake was marginally inversely associated with risk of disabling dementia in women: the multivariate HR (95% CI) for the highest versus lowest quintile was 0.89 (95% CI 0.78–1.02, *P* for trend = 0.050). The highest versus lowest quintile of miso intake was associated with increased risk of disabling dementia: the multivariate HR for the highest versus lowest quintile was 1.16 (95% CI 1.02–1.33, *P* for trend = 0.16). On multiple hypothesis comparison for the interaction term of exposures, natto intake was not significant in women. No soy products were associated with risk of disabling dementia in men. Isoflavone intake was not associated with risk of disabling dementia in both men and women. When we stratified by age, participants aged under 60 years showed a stronger association between natto intake and risk of disabling dementia than those aged 60 years and older in women, but not in men (Tables 3, 4). In women, the multivariate HRs for the highest versus lowest quintile of natto intake were 0.78 (0.59–1.04, *P* for trend = 0.020) for women aged under 60 years and 0.90 (0.77–1.05, *P* for trend = 0.23) for those aged 60 years and older. Other associations between intake of soy products or isoflavones and disabling dementia were similar to the main results in both men and women.

**Table 2:** Multivariate hazard ratios (HRs) and 95% confidence intervals (CIs) for disabling dementia risk according to quintile of total soy product, natto, miso, tofu, and isoflavone intake in men and women

Quintile	First	Second	Third	Fourth	Fifth	Trend *P*
Men						
Total soy product intake^a^, g/day	30.1	52.0	70.9	94.1	141.8	
Person years	35,329	35,254	34,955	35,205	34,523	
No. of cases	363	341	416	404	472	
HR 1 (95% CI)	1.00	0.87 (0.75–1.01)	1.02 (0.88–1.18)	0.95 (0.82–1.10)	1.03 (0.89–1.19)	0.44
HR 2 (95% CI)	1.00	0.93 (0.80–1.08)	1.10 (0.94–1.28)	1.02 (0.87–1.19)	1.07 (0.92–1.24)	0.25
Natto intake^b^, g/day	0.0	4.0	10.9	21.4	42.0	
Person years	38,456	33,987	34,615	34,258	33,951	
No. of cases	557	352	308	325	454	
HR 1 (95% CI)	1.00	1.00 (0.87–1.16)	0.89 (0.76–1.05)	0.84 (0.71–0.99)	0.94 (0.80–1.10)	0.16
HR 2 (95% CI)	1.00	1.02 (0.88–1.18)	0.94 (0.80–1.11)	0.89 (0.75–1.05)	0.999 (0.85–1.17)	0.61
Miso intake^c^, g/day	3.3	10.6	18.1	25.4	36.2	
Person years	35,277	34,816	35,288	35,346	34,540	
No. of cases	403	439	365	340	449	
HR 1 (95% CI)	1.00	0.95 (0.83–1.09)	0.90 (0.77–1.04)	0.82 (0.70–0.95)	1.02 (0.88–1.18)	0.39
HR 2 (95% CI)	1.00	0.99 (0.86–1.14)	0.94 (0.81–1.10)	0.91 (0.77–1.07)	1.11 (0.94–1.31)	0.82
Tofu intake^d^, g/day	9.6	19.8	29.7	44.5	81.6	
Person years	35,164	35,375	35,0640	34,835	34,828	
No. of cases	358	356	385	432	465	
HR 1 (95% CI)	1.00	0.93 (0.80–1.08)	0.93 (0.81–1.08)	0.96 (0.83–1.10)	0.996 (0.87–1.15)	0.87
HR 2 (95% CI)	1.00	0.98 (0.84–1.14)	1.00 (0.86–1.16)	1.01 (0.87–1.17)	1.04 (0.90–1.20)	0.51
Isoflavone intake^e^, g/day	13.4	25.0	35.9	50.1	75.3	
Person years	35,149	35,138	35,054	35,139	34,788	
No. of cases	391	375	372	390	468	
HR 1 (95% CI)	1.00	0.95 (0.83–1.10)	0.99 (0.85–1.15)	0.995 (0.85–1.16)	1.00 (0.86–1.17)	0.85
HR 2 (95% CI)	1.00	0.998 (0.86–1.16)	1.05 (0.90–1.23)	1.08 (0.92–1.27)	1.05 (0.90–1.24)	0.39
Women						
Total soy product intake^a^, g/day	31.0	51.9	70.3	93.0	142.5	
Person years	42,845	43,160	43,264	43,123	42,842	
No. of cases	547	555	561	587	665	
HR 1 (95% CI)	1.00	0.94 (0.84–1.06)	0.90 (0.80–1.02)	0.95 (0.84–1.07)	1.03 (0.92–1.16)	0.55
HR 2 (95% CI)	1.00	0.99 (0.87–1.11)	0.96 (0.84–1.08)	0.999 (0.88–1.13)	1.04 (0.92–1.18)	0.44
Natto intake, g/day	0.2	4.5	11.8	22.1	41.4	
Person years	42,228	43,044	43,612	43,330	43,019	
No. of cases	688	531	481	563	652	
HR 1 (95% CI)	1.00	0.90 (0.80–1.02)	0.82 (0.72–0.93)	0.82 (0.72–0.93)	0.84 (0.73–0.95)	0.002
HR 2 (95% CI)	1.00	0.94 (0.84–1.07)	0.89 (0.78–1.02)	0.88 (0.77–1.00)	0.89 (0.78–1.02)	0.050
Miso intake^c^, g/day	2.7	9.1	14.4	22.1	31.5	
Person years	43,170	43,075	42,786	43,480	42,723	
No. of cases	539	573	588	519	696	
HR 1 (95% CI)	1.00	0.98 (0.87–1.10)	1.01 (0.90–1.14)	0.93 (0.82–1.06)	1.16 (1.03–1.31)	0.13
HR 2 (95% CI)	1.00	1.02 (0.90–1.15)	1.05 (0.93–1.19)	0.97 (0.85–1.11)	1.16 (1.02–1.33)	0.16
Tofu intake^d^, g/day	10.8	21.5	31.8	46.9	81.6	
Person year	42,245	43,439	43,333	43,354	42,862	
No. of cases	623	533	572	564	623	
HR 1 (95% CI)	1.00	0.84 (0.75–0.94)	0.83 (0.74–0.93)	0.84 (0.75–0.94)	0.93 (0.83–1.04)	0.19
HR 2 (95% CI)	1.00	0.91 (0.81–1.03)	0.90 (0.80–1.01)	0.91 (0.81–1.03)	0.98 (0.87–1.11)	0.74
Isoflavone intake^e^, g/day	13.7	25.3	36.2	49.7	74.5	
Person years	42,729	43,059	43,238	43,306	42,901	
No. of cases	571	546	552	565	681	
HR 1 (95% CI)	1.00	0.93 (0.82–1.04)	0.91 (0.80–1.03)	0.89 (0.79–1.01)	0.96 (0.85–1.09)	0.49
HR 2 (95% CI)	1.00	0.96 (0.85–1.09)	0.95 (0.84–1.08)	0.95 (0.84–1.09)	0.98 (0.86–1.12)	0.78

HRs (95% CIs) were derived from Cox proportional hazards regression models

HR 1 was adjusted for age (continuous) and stratified by area. HR 2 was further adjusted for body mass index (< 18.5, 18.5–24.9, 25.0–29.9, 30.0–39.9 kg/m^2^, missing),

smoking status (never, past, < 20 cigarettes/day, ≥ 20 cigarettes/day, missing), alcohol intake (almost never, occasional, < 150, 150–299, ≥ 300 g/week, missing),

history of cancer (yes or no), history of diabetes mellitus (yes or no), medication for hypertension (yes or no) or hypercholesterolemia (yes or no), metabolic equivalents

(quartiles, missing), menopause status (yes, no, missing; women only), health checkup in the past year (yes or no), job status (employed, no, missing), living status (living with others, alone, missing), and intakes of total energy (quintiles), vegetables (quintiles), fruits (quintiles), fish (quintiles) green tea (quintiles) and sodium (quintiles)

Food intake was adjusted for energy intake using the residual method except for total energy and green tea

^a^The sum of tofu (tofu, yushidofu and koya-dofu), natto, miso, deep-fried tofu (abura-age) and soy milk

^b^The first quintile of natto intake included participants who did not eat natto among men. The remaining participants were divided into quartiles and are shown in the second to fifth categories

^c^The intake of miso was calculated from the amount of miso soup

^d^The sum of the amount of tofu, yushidofu and koya-dofu

^e^The sum of the amount of genistein and daidzein

**Table 3:** Multivariate hazard ratios (HRs) and 95% confidence intervals (CIs) for disabling dementia risk according to quintiles of total soy product, natto, miso, tofu and isoflavone intake in men and women aged under 60 years

Quintile	First	Second	Third	Fourth	Fifth	Trend *P*
Men, *n*	2577	2578	2578	2578	2577	
Total soy product intake^a^						
Person years	25,262	25,388	25,368	25,535	25,262	
No. of cases	118	94	120	97	132	
HR (95% CI)	1.00	0.83 (0.62–1.09)	1.01 (0.77–1.33)	0.79 (0.59–1.06)	0.95 (0.72–1.26)	0.74
Natto intake^b^						
Person years	26,996	24,660	24,863	25,072	25,223	
No. of cases	160	106	105	87	103	
HR (95% CI)	1.00	0.98 (0.74–1.28)	1.06 (0.79–1.43)	0.85 (0.62–1.17)	1.06 (0.77–1.44)	0.96
Miso intake^c^						
Person years	25,616	25,272	25,242	25,438	25,247	
No. of cases	113	112	120	94	122	
HR (95% CI)	1.00	0.995 (0.76–1.30)	0.99 (0.76–1.31)	0.81 (0.60–1.10)	1.01 (0.75–1.38)	0.65
Tofu intake^d^						
Person years	25,356	25,184	25,491	25,467	25,316	
No. of cases	105	96	108	127	125	
HR (95% CI)	1.00	0.89 (0.67–1.18)	0.94 (0.71–1.24)	0.98 (0.75–1.29)	0.88 (0.67–1.17)	0.55
Isoflavone intake^e^						
Person years	25,166	25,309	25,201	25,570	25,568	
No. of cases	126	117	112	93	113	
HR (95% CI)	1.00	1.01 (0.78–1.32)	1.03 (0.78–1.37)	0.84 (0.62–1.13)	1.02 (0.76–1.37)	0.76
Women, *n*	2909	2909	2910	2909	2909	
Total soy product intake^a^						
Person years	29,398	29,590	29,701	29,610	29,758	
No. of cases	106	107	114	136	155	
HR (95% CI)	1.00	0.95 (0.72–1.25)	0.91 (0.69–1.20)	1.06 (0.80–1.39)	0.96 (0.73–1.26)	0.99
Natto intake						
Person years	29,422	29,353	29,574	29,753	29,955	
No. of cases	154	121	113	100	130	
HR (95% CI)	1.00	0.87 (0.67–1.13)	0.75 (0.57–0.995)	0.66 (0.50–0.89)	0.78 (0.59–1.04)	0.020
Miso intake^c^						
Person years	29,659	29,737	29,406	29,654	29,601	
No. of cases	94	103	122	124	175	
HR (95% CI)	1.00	1.09 (0.82–1.45)	1.19 (0.90–1.58)	1.12 (0.84–1.51)	1.40 (1.04–1.88)	0.054
Tofu intake^d^						
Person years	29,215	29,730	29,778	29,709	29,625	
No. of cases	126	105	117	124	146	
HR (95% CI)	1.00	0.82 (0.63–1.07)	0.83 (0.64–1.07)	0.81 (0.63–1.06)	0.85 (0.65–1.09)	0.25
Isoflavone intake^e^						
Person years	29,351	29,341	29,658	29,822	29,885	
No. of cases	120	122	117	106	153	
HR (95% CI)	1.00	1.00 (0.77–1.30)	0.91 (0.69–1.20)	0.76 (0.57–1.01)	0.98 (0.74–1.30)	0.50

HRs (95% CIs) were derived from Cox proportional hazards regression models. HR was stratified by area and adjusted for age (continuous), body mass index (< 18.5, 18.5–24.9, 25.0–29.9, 30.0–39.9 kg/m^2^, missing), smoking status (never, past, < 20 cigarettes/day, ≥ 20 cigarettes/day, missing), alcohol intake (almost never, occasional, < 150, 150–299, ≥ 300 g/week, missing), history of cancer (yes or no), history of diabetes mellitus (yes or no), medication for hypertension (yes or no) or hypercholesterolemia (yes or no), metabolic equivalents (quartiles, missing), menopausal status (yes, no, missing; women only), health checkup in the past year (yes or no), job status (employed, no, missing), living status (living with others, alone, missing), and intakes of total energy (quintiles), vegetables (quintiles), fruits (quintiles), fish (quintiles) green tea (quintiles) and sodium (quintiles)

Food intake was adjusted for energy intake using the residual method except for total energy and green tea

^a^The sum of tofu (tofu, yushidofu and koya-dofu), natto, miso, deep-fried tofu (abura-age) and soy milk

^b^The first quintile of natto intake included participants who did not eat natto among men. The remaining participants were divided into quartiles and are shown in the second to fifth categories

^c^The intake of miso was calculated from the amount of miso soup

^d^The sum of the amount of tofu, yushidofu and koya-dofu

^e^The sum of the amount of genistein and daidzein

**Table 4:** Multivariate hazard ratios (HRs) and 95% confidence intervals (CIs) for disabling dementia risk according to quintiles of total soy product, natto, miso, tofu and isoflavone intake in men and women aged 60 years and older

Quintile	First	Second	Third	Fourth	Fifth	Trend *P*
Men, *n*	1220	1221	1221	1221	1220	
Total soy product intake^a^						
Person years	9605	9481	9816	9857	9693	
No. of cases	291	273	306	256	309	
HR (95% CI)	1.00	1.01 (0.85–1.20)	1.16 (0.97–1.38)	0.999 (0.83–1.20)	1.14 (0.96–1.37)	0.18
Natto intake^b^						
Person years	11,460	8975	9448	9266	9304	
No. of cases	397	279	216	259	284	
HR (95% CI)	1.00	1.05 (0.89–1.25)	0.85 (0.69–1.03)	0.99 (0.81–1.21)	1.01 (0.83–1.22)	0.84
Miso intake^c^						
Person years	9381	9734	9915	9817	9605	
No. of cases	315	311	249	250	310	
HR (95% CI)	1.00	0.97 (0.83–1.14)	0.86 (0.72–1.03)	0.96 (0.79–1.15)	1.11 (0.91–1.34)	0.91
Tofu intake^d^						
Person years	9531	9890	9543	9752	9736	
No. of cases	278	285	284	288	300	
HR (95% CI)	1.00	1.07 (0.91–1.27)	1.09 (0.92–1.30)	1.08 (0.91–1.28)	1.14 (0.96–1.35)	0.17
Isoflavone intake^e^						
Person years	9675	9467	9800	9668	9842	
No. of cases	301	273	295	273	293	
HR (95% CI)	1.00	1.02 (0.86–1.21)	1.12 (0.94–1.34)	1.10 (0.91–1.32)	1.12 (0.92–1.35)	0.20
Women, *n*	1582	1582	1582	1582	1582	
Total soy product intake^a^						
Person years	12,900	13,555	13,637	13,695	13,390	
No. of cases	520	451	432	422	472	
HR (95% CI)	1.00	0.93 (0.82–1.06)	0.90 (0.79–1.04)	0.91 (0.79–1.04)	1.04 (0.91–1.20)	0.67
Natto intake						
Person years	13,006	13,283	13,720	13,591	13,577	
No. of cases	505	480	400	462	450	
HR (95% CI)	1.00	0.97 (0.85–1.11)	0.87 (0.75–1.02)	0.97 (0.83–1.13)	0.90 (0.77–1.05)	0.23
Miso intake^c^						
Person years	13,184	13,431	13,453	13,819	13,290	
No. of cases	498	457	450	406	486	
HR (95% CI)	1.00	0.99 (0.87–1.13)	0.98 (0.86–1.13)	0.91 (0.79–1.05)	1.08 (0.92–1.25)	0.94
Tofu intake^d^						
Person years	12,839	13,588	13,674	13,608	13,468	
No. of cases	519	446	445	446	441	
HR (95% CI)	1.00	0.92 (0.81–1.05)	0.92 (0.80–1.04)	0.98 (0.86–1.12)	0.97 (0.85–1.12)	0.90
Isoflavone intake^e^						
Person years	13,089	13,360	13,605	13,690	13,432	
No. of cases	494	469	442	432	460	
HR (95% CI)	1.00	1.03 (0.90–1.17)	0.98 (0.85–1.13)	0.99 (0.85–1.14)	1.03 (0.89–1.19)	0.84

HRs (95% CIs) were derived from Cox proportional hazards regression models. HR was stratified by area and adjusted for age (continuous), body mass index (< 18.5, 18.5–24.9, 25.0–29.9, 30.0–39.9 kg/m^2^, missing), smoking status (never, past, < 20 cigarettes/day, ≥ 20 cigarettes/day, missing), alcohol intake (almost never, occasional, < 150, 150–299, ≥ 300 g/week, missing), history of cancer (yes or no), history of diabetes mellitus (yes or no), medication for hypertension (yes or no) or hypercholesterolemia (yes or no), metabolic equivalents (quartiles, missing), menopausal status (yes, no, missing; women only), health checkup in the past year (yes or no), job status (employed, no, missing), living status (living with others, alone, missing) and intakes of total energy (quintiles), vegetables (quintiles), fruits (quintiles), fish (quintiles) green tea (quintiles) and sodium (quintiles)

Food intake was adjusted for energy intake using the residual method except for total energy and green tea

^a^The sum of tofu (tofu, yushidofu and koya-dofu), natto, miso, deep-fried tofu (abura-age) and soy milk

^b^The first quintile of natto intake included participants who did not eat natto among men. The remaining participants were divided into quartiles and are shown in the second to fifth categories

^c^The intake of miso was calculated from the amount of miso soup

^d^The sum of the amount of tofu, yushidofu and koya-dofu

^e^The sum of the amount of genistein and daidzein

The association between intake of soy products or isoflavones and disabling dementia was similarly observed when we analyzed the data for cases of disabling dementia with or without a history of stroke in both men and women (Supplemental Tables 1 and 2). The multivariate HRs for the highest versus lowest quintile of natto intake were 0.75 (0.50–1.11, *P* for trend = 0.21) for cases of disabling dementia with a history of stroke and 0.81 (0.65–1.01, *P* for trend = 0.017) for those without a history of stroke in women (Supplemental Table 2). In multistate model analyses assessing the effect of natto intake on the transitions between the various states, the multivariate-adjusted HRs associated with the highest quintile of natto intake in women were 0.78 (0.64–0.96) for dementia occurrence in healthy individuals; 1.01 (0.69–1.47) for stroke occurrence in healthy individuals; and 0.54 (0.23–1.27) for dementia occurrence in individuals who previously suffered an incident stroke event (Supplemental Fig. 2).

## Discussion

In this study, we found that intake of total soy products and isoflavones was not associated with the risk of disabling dementia in both men and women. For individual soy products, natto intake was marginal inversely associated with risk of disabling dementia in women, whereas no soy product was associated with this risk in men. This is the first cohort study to reveal an association between intake of total soy, individual soy products or isoflavones and risk of incident disabling dementia in middle-aged Japanese men and women.

We showed a marginal inverse association between natto intake and disabling dementia in women. Natto contains isoflavone, whose intake has shown protective effects on cognitive function and AD via a reduction in the level of inflammation and oxidative stress [4]. In addition, isoflavone intake induced the degradation of amyloid-β protein via peroxisome proliferator-activated receptor gamma/apolipoprotein E activation, and decreased tau phosphorylation, which causes the formation of neurofibrillary tangles [5]. However, given the lack of any association between other soy products and isoflavone and disabling dementia in this study, the mechanism likely relates to the unique components and characteristics of natto. Nattokinase, an enzyme contained in the sticky component of natto, shows amyloid-degrading ability in vitro [18]. In addition, oral administration of nattokinase in a rat model of AD showed a significant decrease in brain levels of apoptotic and inflammatory factors, and an increase in brain-derived neurotrophic factor and insulin-like growth factor-1 levels when compared with untreated AD-induced rats [19]. In Alzheimer disease-induced rats, a high dose of nattokinase resulted in the disappearance of amyloid plaques in the hippocampus [19]. Natto is also rich in polyamine [15]. Drosophila fed with polyamine were protected against age-induced memory impairment [17]. Natto intake may protect the hippocampus against β-amyloid-induced neuronal damage. In previous cohort studies [20, 21], furthermore, natto intake was associated with a decreased risk of hypertension and stroke, and may have prevented vascular dementia. Regarding other reasons, participants who responded that they did not or only rarely consumed natto may have had poor masticatory function. Poor masticatory function has been associated with risk of dementia in elderly people [36]. Most soy products have a soft texture, including tofu (soybean curd) and miso (soybean paste), but natto is made by fermenting steamed whole soybeans using Bacillus subtilis [37]. A previous study reported that masticatory function increased regional cerebral blood flow [38, 39]. Moriya et al. reported that poor self-assessed masticatory status was associated with poor verbal and visual immediate explicit memory function [40]. To date, only one prospective study from Japan reported no association between natto intake and cognitive function in 403 men and 373 women aged 60–81 years [8]. When we stratified by age, natto intake was significantly inversely associated with disabling dementia in women aged under 60 years, but not in those aged 60 years and older (Tables 3, 4). We considered that this study design allowed us to eliminate the effect of aging or cognitive impairment on dietary intake, because exposure of soy product intake was obtained in middle-aged participants, especially for under aged 60 years. In this study, there was a higher percentage of medical history in participants aged 60 years and older than in those under 60 years in women: respective proportions were 32.4% and 12.8% for medication for hypertension; 13.9% and 5.2% for medication for hypercholesterolemia; and 5.3% and 2.6% for a history of diabetes mellitus. Although we adjusted for these medical histories, other risk factors affected disabling dementia risk more strongly than these variables, and may have attenuated the association between natto intake and dementia risk in older participants. Although the cause of this difference is not clear, the effect of natto intake in reducing risk of disabling dementia may be stronger in younger than older people.

We also found that the inverse association between natto intake and risk of disabling dementia was found only among women. Although the prevalence of smokers and drinkers was higher among men than women, we found no change in the results by smoking and heavy drinking status when we stratified by former/current or never smokers and over or under 300 ethanol g/week drinkers in men. Moreover, although we adjusted for smoking or drinking status, we considered that other residual confounding remained. The results of this study are similar to those of a previous study in which soy isoflavone intake was associated with decreased cognitive impairment in women only, although it was not associated with natto [8]. Accordingly, the meaning of the sex difference in the results remains unclear and further studies are needed.

In this study, only the highest quintile of miso intake was associated with an increased risk of disabling dementia, and no significant trend was observed among women. The salt content of miso is high compared to other soy products, particularly when considered with regard to frequency of intake. While high salt intake increases the risk of stroke [41], considered a risk factor of dementia [42], our previous prospective study reported that miso intake was not associated with incidence of high blood pressure [20] or cardiovascular disease [21]. The reason for the tendency toward a positive association between miso intake and risk of disabling dementia is unclear, and further studies are needed.

A prospective study showed that intake of total soy product or isoflavone was not associated with cognitive function in Chinese men and women [9]. Consistent with that study, we also found no association between intake of total soy products or isoflavones and risk of disabling dementia in either men or women. Two prospective studies reported that soy product intake was associated with decreased risk of cognitive function [7, 8]. An et al. found that soy product intake was inversely associated with risk of cognitive impairment in a cognitively intact Chinese population aged 80 years and older at baseline [7]; however, this finding considered attributable to selection bias. Another study from Japan reported that intake of total soy products, soybeans and isoflavones was associated with a significantly decreased risk of cognitive impairment in women [8]. According to Ozawa et al. [10], a healthy dietary pattern which included soy products was associated with decreased dementia risk in participants aged 60 years and older, but this result might have been affected by soy and other healthy foods. Regarding isoflavone intake, we did not include soybeans, which are richer in isoflavones than tofu and miso [32], in total soy product intake, nor glycitin (an isoflavone) in isoflavone intake, despite its inclusion in a previous study [8]. Although a previous meta-analysis study reported that isoflavone improved cognitive function in randomized control studies, only two of the included studies were from Asia [6]. In addition, because the dietary intake of isoflavone is higher in Asia than in Western countries [43–45], further studies are needed to confirm its effects on cognitive function in Asian people.

The strengths of this study include its use of middle-aged participants at the survey point of exposure, its large sample size, long follow-up, use of a validated FFQ and adjustment for potentially important confounding factors, including smoking status, alcohol intake, and other food items. In addition, disabling dementia was validated by a clinical assessment for dementia. The long follow-up period is likely to allow estimation of a robust association between soy product intake and risk of disabling dementia. Despite these strengths, several limitations warrant discussion. First, disabling dementia was originally established for the Japanese system, and this outcome is not diagnosed by psychiatrists. However, the validation of disabling dementia criteria was confirmed by comparison with the clinical diagnoses of neuropsychiatrists with moderate sensitivity and with high specificity [25], and grades of cognitive disability were well-correlated with the MMSE score [26]. Second, we could not classify disabling dementia into Alzheimer's or vascular types. Although the number of cases was relatively small, the association of total soy, individual soy product and isoflavone intake was similarly observed with disabling dementia regardless of a history of stroke in both men and women (Supplemental Tables 1 and 2). Therefore, natto intake may protect against disabling dementia regardless of dementia type in women. Based on the multistate model analyses, we found no evidence to suggest that the effect of natto intake on dementia occurrence could be partly due to an effect of natto intake on the occurrence of stroke in women (Supplemental Fig. 2). Third, although participants may have changed dietary habits during follow-up, soy product intake was assessed only one time. Any such dietary change might have attenuated the association shown in the present study. However, Spearman rank correlation coefficients of the estimated energy-adjusted intake of soy products between two FFQs administered at a one-year interval were moderate: 0.64 for men and 0.67 for women in cohort I; and 0.57 and 0.44 in cohort II [30, 46]. Fourth, we did not have information on cognitive function or dementia status among participants at the follow-up start point. However, exposure information was collected around 10 years before the incidence of disabling dementia. In addition, similar results were obtained when analysis was limited to data collected around 5 years before the incidence of disabling dementia, and even after the exclusion of subjects who needed caregivers in activities of daily living, natto intake was still inversely associated with the risk of disabling dementia in women: the multivariate HR (95% CI) for the highest versus lowest quintile of natto intake was 0.84 (0.72–0.98, *P* for trend = 0.031) (data not shown in Table). Moreover, considering that the onset of disabling dementia occurs over a long time, the first year of registration may include participants who already had dementia. The results did not substantially change when cases diagnosed within the first year of follow-up were excluded in men. The marginal inverse association between natto intake and disabling dementia risk was attenuated: the multivariate HR (95% CI) for the highest versus lowest quintile was 0.93 (0.81–1.07, *P* for trend = 0.19), but women aged under 60 years still showed a marginal inverse association with risk, with a multivariate HR (95% CI) for the highest versus lowest quintile of 0.82 (0.62–1.10, *P* for trend = 0.054) in women aged under 60 years (data not shown in Table). Furthermore, education level also may be a confounding factor, but we did not adjust this for all subjects, because we only had information about education level in cohort I (*n* = 19,148, Yokote, Saku and Uruma areas). Nevertheless, when we adjusted for education level (junior high school or less, high school, and university or higher education, missing) in cohort I participants only, the inverse association between natto intake and disabling dementia risk was not changed (data not shown in Table). Fifth, the risk of disabling dementia with the lowest quintile of natto intake might be overestimated because some participants were prescribed warfarin and were prohibited from eating natto because of its high concentration of vitamin K [47]. These participants were likely to have had atrial fibrillation or valvular disease which in turn likely enhanced the risk of stroke and vascular dementia. However, when we excluded participants with a history of cardiovascular disease from 5-year follow-up questionnaires (stroke, myocardial infarction or angina, *n* = 460 excluded), natto intake still showed an inverse association with the risk of disabling dementia in women: the multivariate HR (95% CI) for the highest versus lowest quintile was 0.86 (0.75–0.99, *P* for trend = 0.011).

In conclusion, although further studies are warranted to confirm the generalization of these results, natto intake may contribute to reducing the risk of disabling dementia in women.

## Supplementary Information

Below is the link to the electronic supplementary material.

Supplementary file1 (PDF 330 KB)

## Abbreviations

PHC: Public health center
FFQ: Food frequency questionnaire
HR: Hazard ratio
CI: Confidence interval
