# Supplementary material for: Soy product intake and risk of incident disabling dementia: the JPHC Disabling Dementia Study
Source: Eur J Nutr. 2022 Jul 5;61(8):4045–57. doi: 10.1007/s00394-022-02937-5 (PMC9596534; doi:10.1007/s00394-022-02937-5)
Supplement: Supplementary file 1 — Supplementary file1 (PDF 330 KB) [file 394_2022_2937_MOESM1_ESM.pdf]

Online Supplemental Material

Title: Soy product intake and risk of incident disabling dementia: the JPHC Disabling Dementia Study.

Journal: European Journal of Nutrition

Author names: Utako Murai, Norie Sawada, Hadrien Charvat, Manami Inoue, Nobufumi Yasuda, Kazumasa Yamagishi, Shoichiro Tsugane, for the JPHC Study Group

Corresponding author: Norie Sawada, M.D., Ph.D.

Division of Cohort Research, National Cancer Center Institute for Cancer Control

E-mail: nsawada@ncc.go.jp

Supplemental Figure 1. Participant flow chart.

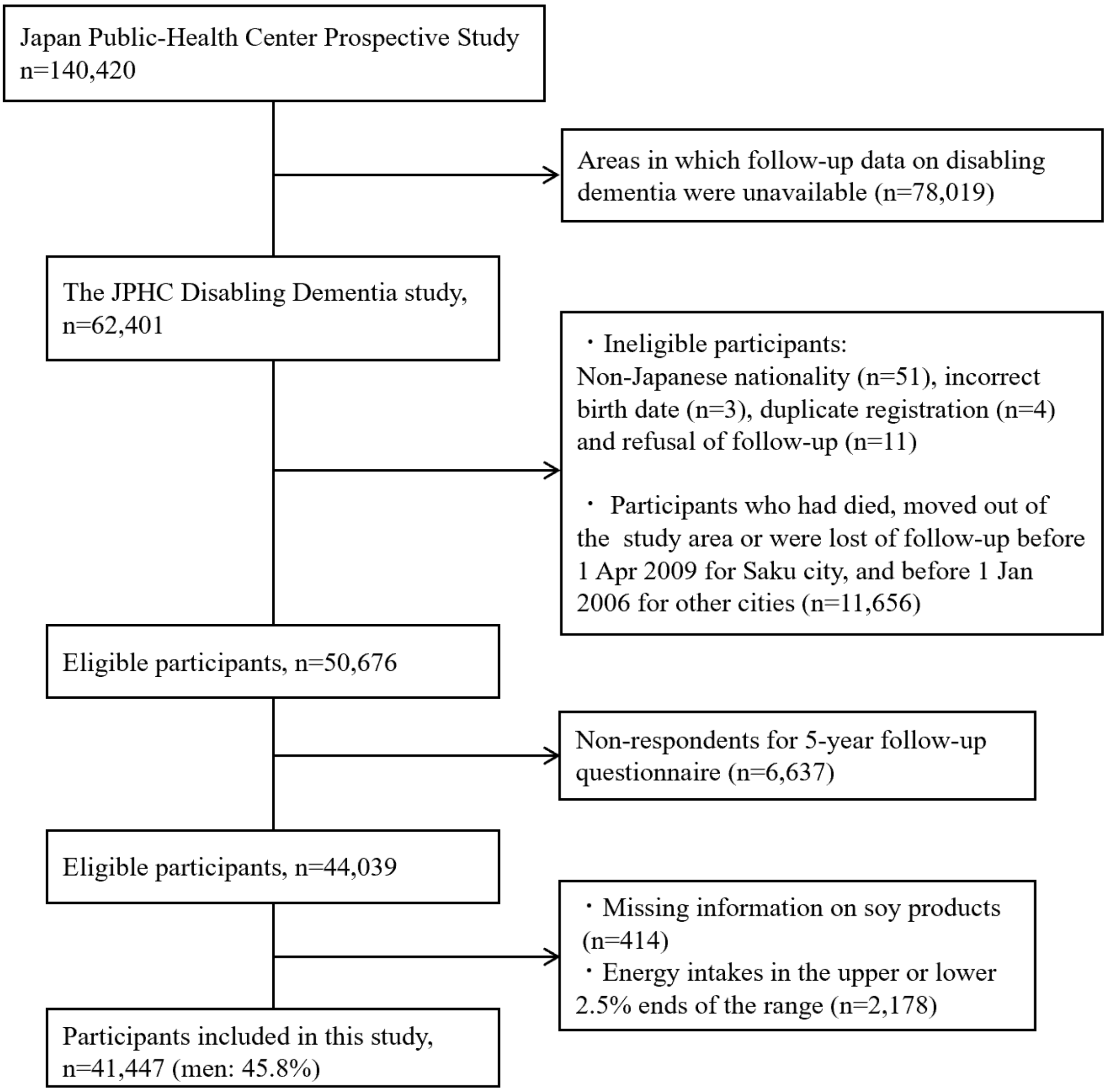

Supplemental Figure 2. Multistate models for the role of natto intake in transitions to incident stroke and disabling dementia in women.

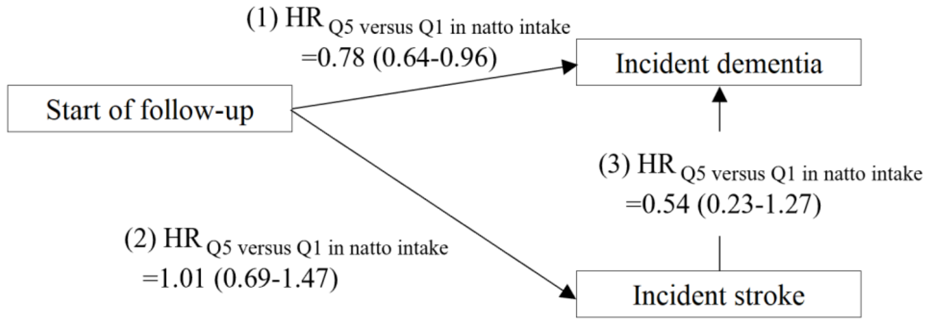

HRs (95%CIs) were derived from multistate models, which were used to simultaneously estimate risk associated with soy product intake in three transitions: (1) from healthy state to incident dementia in those free of incident stroke; (2) from healthy state to incident stroke; and (3) from incident stroke to incident dementia.

Supplemental Table 1. Multivariate hazard ratios (HRs) and 95% confidence intervals (CIs) for disabling dementia risk according to quintiles of total soy product, natto, miso, tofu and isoflavone intake by cases of disabling dementia with or without a history of stroke in men[a].

Quintile	First	Second	Third	Fourth	Fifth	Trend p
Total soy product intake[b]						
Person years	19650	18768	18513	18662	18268	
Dementia with a history of stroke						
No. of cases	64	48	74	68	89	
HR (95% CI)	1.00	0.85 (0.58-1.25)	1.26 (0.88-1.80)	1.12 (0.77-1.63)	1.26 (0.88-1.80)	0.11
Dementia without a history of stroke						
No. of cases	134	103	112	132	128	
HR (95% CI)	1.00	0.76 (0.59-0.995)	0.83 (0.63-1.09)	0.95 (0.73-1.24)	0.78 (0.60-1.03)	0.24
Natto intake[c]						
Person years	20049	17434	18148	18557	19673	
Dementia with a history of stroke						
No. of cases	99	57	40	67	80	
HR (95% CI)	1.00	0.90 (0.63-1.29)	0.73 (0.48-1.12)	1.04 (0.70-1.54)	0.95 (0.64-1.39)	0.92
Dementia without a history of stroke						
No. of cases	167	105	92	87	158	
HR (95% CI)	1.00	1.14 (0.87-1.48)	1.09 (0.81-1.48)	0.85 (0.62-1.16)	1.11 (0.84-1.47)	0.84
Miso intake[d]						
Person years	20229	19447	18499	18026	17659	
Dementia with a history of stroke						
No. of cases	78	83	61	55	66	
HR (95% CI)	1.00	1.04 (0.75-1.43)	0.91 (0.64-1.30)	0.97 (0.66-1.42)	0.97 (0.65-1.44)	0.75
Dementia without a history of stroke						
No. of cases	149	151	107	91	111	
HR (95% CI)	1	0.96 (0.76-1.21)	0.88 (0.68-1.15)	0.84 (0.63-1.12)	0.85 (0.63-1.15)	0.20
Tofu intake[e]						
Person years	19754	19077	18752	18357	17921	
Dementia with a history of stroke						
No. of cases	55	60	74	59	95	
HR (95% CI)	1.00	1.14 (0.78-1.65)	1.40 (0.97-2.00)	0.96 (0.66-1.42)	1.48 (1.04-2.11)	0.065
Dementia without a history of stroke						
No. of cases	137	97	113	128	134	
HR (95% CI)	1	0.77 (0.59-1.01)	0.79 (0.61-1.02)	0.80 (0.62-1.04)	0.85 (0.66-1.10)	0.29
Isoflavone intake[f]						
Person years	18767	18273	18404	18958	19459	
Dementia with a history of stroke						
No. of cases	62	61	54	78	88	
HR (95% CI)	1.00	1.12 (0.77-1.62)	1.07 (0.72-1.58)	1.50 (1.03-2.19)	1.34 (0.91-1.97)	0.065
Dementia without a history of stroke						
No. of cases	124	120	106	119	140	
HR (95% CI)	1.00	1.01 (0.78-1.31)	0.98 (0.74-1.30)	1.00 (0.75-1.33)	0.88 (0.66-1.18)	0.41

HRs (95%CIs) were derived from Cox proportional hazards regression models. HR was stratified by area and adjusted for age (continuous), body mass index (<18.5, 18.5-24.9, 25.0-29.9, 30.0-39.9 kg/m^2, missing), smoking status (never, past, <20 cigarettes/day, ≥20 cigarettes/day, missing), alcohol intake (almost never, occasional, <150, 150-299, ≥300 g/week, missing), history of cancer (yes or no), history of diabetes mellitus (yes or no), medication for hypertension (yes or no) or hypercholesterolemia (yes or no), metabolic equivalents (quartiles, missing), health checkup in the past year (yes or no), job status (employed, no, missing), living status (living with others, alone, missing) and intakes of total energy (quintiles), vegetables (quintiles), fruits (quintiles), fish (quintiles), green tea (quintiles) and sodium (quintiles).

[a] Food intake was adjusted for energy intake using the residual method except for total energy and green tea.

[b] The sum of tofu (tofu, yushidofu and koya-dofu), natto, miso, deep-fried tofu (abura-age) and soy milk.

[c] The first quintile of natto intake included participants who did not eat natto. The remaining participants were divided into quartiles and are shown in the second to fifth categories.

[d] The intake of miso was calculated from the amount of miso soup.

[e] The sum of the amount of tofu, yushidofu and koya-dofu.

[f] The sum of the amount of genistein and daidzein.

Supplemental Table 2. Multivariate hazard ratios (HRs) and 95% confidence intervals (CIs) for disabling dementia risk according to quintiles of total soy product, natto, miso, tofu and isoflavone intake by cases of disabling dementia with or without a history of stroke in women[a].

Quintile	First	Second	Third	Fourth	Fifth	Trend p
Total soy product intake[b]						
Person years	23540	23139	22598	22216	21293	
Dementia with a history of stroke						
No. of cases	52	62	54	83	65	
HR (95% CI)	1.00	1.24 (0.85-1.82)	1.03 (0.69-1.55)	1.64 (1.12-2.38)	1.15 (0.78-1.71)	0.26
Dementia without a history of stroke						
No. of cases	223	190	182	176	214	
HR (95% CI)	1.00	0.88 (0.72-1.08)	0.86 (0.70-1.06)	0.84 (0.68-1.04)	0.97 (0.79-1.19)	0.69
Natto intake						
Person years	21828	21496	22326	23242	23893	
Dementia with a history of stroke						
No. of cases	74	56	47	69	70	
HR (95% CI)	1.00	0.86 (0.59-1.26)	0.76 (0.50-1.16)	0.89 (0.60-1.33)	0.75 (0.50-1.11)	0.21
Dementia without a history of stroke						
No. of cases	266	169	145	169	236	
HR (95% CI)	1.00	0.82 (0.66-1.01)	0.78 (0.62-0.99)	0.68 (0.54-0.86)	0.81 (0.65-1.01)	0.017
Miso intake[c]						
Person years	23730	23788	22723	21623	20922	
Dementia with a history of stroke						
No. of cases	59	63	61	56	77	
HR (95% CI)	1.00	1.07 (0.75-1.55)	0.99 (0.68-1.44)	1.02 (0.68-1.53)	1.07 (0.71-1.61)	0.85
Dementia without a history of stroke						
No. of cases	196	208	217	158	206	
HR (95% CI)	1.00	1.02 (0.83-1.25)	1.17 (0.95-1.44)	0.96 (0.76-1.21)	1.08 (0.85-1.37)	0.58
Tofu intake[d]						
Person years	23809	23245	22816	22124	20792	
Dementia with a history of stroke						
No. of cases	70	55	57	67	67	
HR (95% CI)	1.00	0.95 (0.66-1.37)	0.92 (0.64-1.32)	1.21 (0.85-1.72)	1.17 (0.82-1.67)	0.24
Dementia without a history of stroke						
No. of cases	256	180	192	168	189	
HR (95% CI)	1.00	0.82 (0.68-1.00)	0.82 (0.67-0.99)	0.78 (0.63-0.95)	0.88 (0.72-1.07)	0.11
Isoflavone intake[e]						
Person years	22393	22355	22390	22897	22752	
Dementia with a history of stroke						
No. of cases	56	52	62	77	69	
HR (95% CI)	1.00	0.95 (0.64-1.40)	1.13 (0.76-1.68)	1.31 (0.89-1.93)	0.97 (0.65-1.45)	0.72
Dementia without a history of stroke						
No. of cases	229	166	178	186	226	
HR (95% CI)	1.00	0.76 (0.62-0.94)	0.84 (0.67-1.04)	0.83 (0.67-1.03)	0.85 (0.68-1.05)	0.22

HRs (95%CIs) were derived from Cox proportional hazards regression models. HR was stratified by area and adjusted for age (continuous), body mass index (<18.5, 18.5-24.9, 25.0-29.9, 30.0-39.9 kg/m^2, missing), smoking status (never, past, <20 cigarettes/day, ≥20 cigarettes/day, missing), alcohol intake (almost never, occasional, <150, 150-299, ≥300 g/week, missing), history of cancer (yes or no), history of diabetes mellitus (yes or no), medication for hypertension (yes or no) or hypercholesterolemia (yes or no), metabolic equivalents (quartiles, missing), menopause (yes, no, missing), health checkup in the past year (yes or no), job status (employed, no, missing), living status (living with others, alone, missing) and intakes of total energy (quintiles), vegetables (quintiles), fruits (quintiles), fish (quintiles), green tea (quintiles) and sodium (quintiles).

[a] Food intake was adjusted for energy intake using the residual method except for total energy and green tea.

[b] The sum of tofu (tofu, yushidofu and koya-dofu), natto, miso, deep-fried tofu (abura-age) and soy milk.

[c] The intake of miso was calculated from the amount of miso soup.

[d] The sum of the amount of tofu, yushidofu and koya-dofu.

[e] The sum of the amount of genistein and daidzein.
